# Three-dimensional high-content imaging of unstained soft tissue with subcellular resolution using a laboratory-based X-ray microscope

**DOI:** 10.1073/pnas.2525239123

**Published:** 2026-03-17

**Authors:** Michela Esposito, Alberto Astolfo, Yang Zhou, Ian Buchanan, Alexei Teplov, John Ciaran Hutchinson, Marco Endrizzi, Alexandra Egido Vinogradova, Olga Makarova, Ralu Divan, Cha-Mei Tang, Yukako Yagi, Peter D. Lee, Claire L. Walsh, Joseph D. Ferrara, Alessandro Olivo

**Affiliations:** ^a^Department of Medical Physics and Biomedical Engineering, University College London, London WC1E 6BT, United Kingdom; ^b^Department of Mechanical Engineering, University College London, London WC1E 6BT, United Kingdom; ^c^Department of Pathology and Lab Medicine, Memorial Sloan Kettering Cancer Center, New York 10065, NY; ^d^Department of Histopathology, Great Ormond Street Hospital for Children National Health Service Foundation Trust, London WC1N 1EH, United Kingdom; ^e^X-ray microscopy and tomography lab, The Francis Crick Institute, London NW1 1AT, United Kingdom; ^f^Rigaku Americas, The Woodlands, TX 77381; ^g^Creatv MicroTech Inc., Chicago, IL 60612; ^h^Center for Nanoscale Materials, Argonne National Laboratory, Lemont, IL 60439; ^i^Creatv MicroTech Inc., Potomac, MD 20854

**Keywords:** X-ray phase contrast CT, X-ray dark field imaging, X-ray microscopy, 3D virtual histology, cell imaging

## Abstract

With increasing interest in studying biological systems across spatial scales—from centimeters down to nanometers—histology continues to be the gold standard for tissue imaging at cellular resolution, providing an essential bridge between macroscopic and nanoscopic analysis. However, its inherently destructive and two-dimensional nature limits its ability to capture the full three-dimensional complexity of tissue architecture. Here, we show that phase-contrast X-ray microscopy can enable three-dimensional virtual histology with subcellular resolution. This technique provides direct quantification of electron density without restrictive assumptions, allowing for direct characterization of cellular nuclei in a standard laboratory setting. By combining high spatial resolution and soft tissue contrast, with automated segmentation of cell nuclei, we demonstrated virtual Hematoxylin and Eosin (H&E) staining using machine learning-based style transfer, yielding volumetric datasets compatible with existing histopathological analysis tools. Furthermore, by integrating electron density and the sensitivity to nanometric features of the dark field contrast channel, we achieve stain-free, high-content imaging capable of distinguishing nuclei and extracellular matrix.

## Significance

Three-dimensional analysis of biological tissue is crucial for linking tissue morphology to cellular function. While conventional histology remains the gold standard for visualizing tissue architecture at subcellular resolution, it is limited by its destructive, two-dimensional nature and anisotropic resolution, hindering its use in capturing full three-dimensional representations of tissue volumes. Here, we demonstrate that phase-contrast X-ray microscopy enables nondestructive, three-dimensional virtual histology of unstained liver tissue with subcellular resolution. By automatically detecting cells’ nuclei over three-dimensional tissue volumes and quantifying relevant parameters (e.g. electron density, nuclear morphology), this instrument has the potential to provide capabilities in biomedicine linking volumetric tissue architecture with cell-scale quantification.

Biomedicine is increasingly dependent on multimodal and multiscale imaging approaches to bridge vastly different length scales—from whole-organ imaging using standard clinical techniques, down to molecular level with -omics. Conventional histology remains the method of choice to connect macroscopic and nanoscopic domains, offering subcellular resolution and functional characterization of soft tissues through a diverse array of staining protocols. However, its inherently destructive nature and the introduction of tissue processing artifacts significantly limit its applicability to three-dimensional imaging (1). Beyond histology, optical microscopy continues to be the gold standard for probing the microscale, but its efficacy quickly diminishes in thick tissue samples, due to the need for optical clearing (2).

The demand for maintaining subcellular resolution across increasingly thick samples has spurred growing interest in more penetrating probes, such as X-rays. Conventional X-ray imaging modalities, however, lack sufficient soft tissue contrast, making cellular structures difficult to resolve without staining, due to the weak contribution of the imaginary part (β) of the complex refractive index (n=1−δ+iβ) in the hard X-ray regime. Conversely, the unit decrement of the real part of the refractive index (δ) dominates at these energies and can be exploited by indirect measurement of the phase-shifts of waves traveling through the sample. The combination of X-rays’ penetrating power and the enhanced soft tissue contrast enabled by phase-sensitive techniques, combined with the penetrating power of X-rays, enabled significant breakthroughs with the use of synchrotron radiation (3–11).

Notably, synchrotron-based phase-contrast imaging has enabled a functional and multiscale investigation of the brain activity with subcellular resolution (12), the identification of pathological states of the brain (Alzheimer’s) at the cells’ nuclear level (13), a multimodal and multiscale visualization of neurodegenerative state of the brain (14), visualization of an entire mouse brain with submicron voxels (15), single cell imaging combined with confocal microscopy to identify inactive regions of the nuclear genetic material (16). Whole organs can now be imaged with cellular resolution at synchrotron facilities, using a nondestructive hierarchical scan protocol (17) enabling multiscale imaging of a whole adult heart (18) and of a human placenta (19). Crucially, Li et al. (20) showed that routine histochemical, immunohistochemical, DNA, and RNA analysis are compatible with synchrotron imaging, suggesting the feasibility of linking morphology with the molecular domain for tissue samples.

The translation of phase-contrast imaging techniques from specialized synchrotron facilities to the laboratory environment, however, presents important challenges when subcellular resolution in unstained tissue is required. The potential of phase-contrast laboratory-based imaging at tissue scale has been shown in refs. 21–23, enabling accurate identification of tumor margins, closely matching standard histological findings. However, these systems feature a resolution limit in order of 10 to 20 μm, insufficient for cellular resolution. Multiple efforts have been reported that target cellular or subcellular resolution in tissue samples. Vagberg et al. (24) reported 3D imaging of unstained human coronary arteries and, while cellular resolution was claimed, only large (≈50 μm) adipose cells or clusters of foam cells were visible. A comparative study of synchrotron and laboratory-based imaging of diseased human hearts is reported in ref. 25, showing how the segmentation of the vascular network allows identification of different pathologies. Cellular resolution, however, is achieved only in the case of synchrotron imaging.

Cellular resolution with a laboratory-based set-up has been demonstrated in refs. 26 and 27 for renal tissue with eosin staining (a stain typically used in pathology), followed by critical point drying. The chosen tissue processing, however, limits possible downstream investigations (e.g. molecular analysis) and the choice of counterstains in histology. This strongly hinders the possibility of integrating this system in a multimodal imaging workflow. Different tissue processing approaches were tested for brain imaging in ref. 28, with samples scanned both with synchrotron radiation and with a laboratory-based CT scanner. While neurons and axons were visible with the laboratory-based system with all tissue preparation protocols, cells were only visible when samples had been stained with heavy metals (osmium tetroxide). Digital histology of unstained kidney glomeruli with an X-ray source has been reported in ref. 29. Although cellular-level imaging is claimed, the authors conclude that cell-like features reported in the article cannot be confirmed to be cells due to the lack of suitable verification (e.g. matching histology). Reichmann et al. (30, 31) report how synchrotron and laboratory-based phase-contrast imaging can enable the detection of pulmonary pathologies. However, cellular resolution was only achieved for synchrotron-based imaging. Cellular visualization for brain tissue has been reported in refs. 32–34. Segmentation and a quantitative analysis of large and ramified Purkinje cells, as well as of smaller cells in the granular and molecular layers, are reported for both synchrotron and laboratory setup (33). It has to be noted, however, that quantitative parameters for the segmented nuclei (e.g. electron density, radii, nearest neighbor distance) derived from laboratory-based measurements do not show a close agreement with the gold-standard synchrotron measurements. The laboratory-based imaging systems referenced above are all based on propagation-based approaches (35), providing access to a single, nonquantitative contrast channel. When propagation-based approaches are used, even in the synchrotron environment, electron density information extracted from tomographic scans rely on the restrictive hypothesis of sample homogeneity (i.e. constituted by at most two different materials) (36) and calibrated with respect to the substrate material of the sample (37), which requires an additional assumption on its chemical composition.

In this work, we report the demonstration of nondestructive fully quantitative three-dimensional imaging of unstained liver tissue, using a laboratory-based phase-contrast X-ray imaging system. Making use of quasi-monochromatic radiation, we ensure direct and quantitative access to electron density of the microscopy scale. Liver tissue, which typically features a high cell density, represents the optimal benchmark for demonstrating the capability of this system to detect the faint electron density difference needed to resolve cellular components. At the microscopic level, the liver can also show pathological states such as vesicular fat metamorphosis and changes in the collagen arrangement in the extracellular matrix (ECM) leading to fibrosis. In this article, we show how the direct measurement of electron density allows the visualization of hepatocytes nuclei over a mm^3^ of unstained Formalin-Fixed-Paraffin Embedded (FFPE) liver tissue, extracted from a conventional histology cassette. The achieved resolution (real 1 μm, as opposed to voxel size typically quoted for commercial CT scanners) and contrast facilitated the automatic segmentation of cells’ nuclei and quantification of morphological parameters. The image quality of the measured electron density maps, combined with the accuracy of the segmentation workflow, enabled the use of a Machine Learning (ML) algorithm for converting measured three-dimensional electron density maps into three-dimensional H&E histology volumes.

The imaging system used in this work allows for the retrieval of three complementary contrast channels (38). In addition to phase (proportional to electron density), of particular interest is the dark field or Ultra-Small Angle X-ray Scattering (USAXS) that can identify the presence of ensembles of features below the system resolution (39), i.e. in the nanoscale. Here, we demonstrate that, combining electron density and dark field maps, we can identify collagen fibrils in the ECM delivering high-content imaging.

## Results

### Three-Dimensional Cell Visualization.

**Fig. 1.**
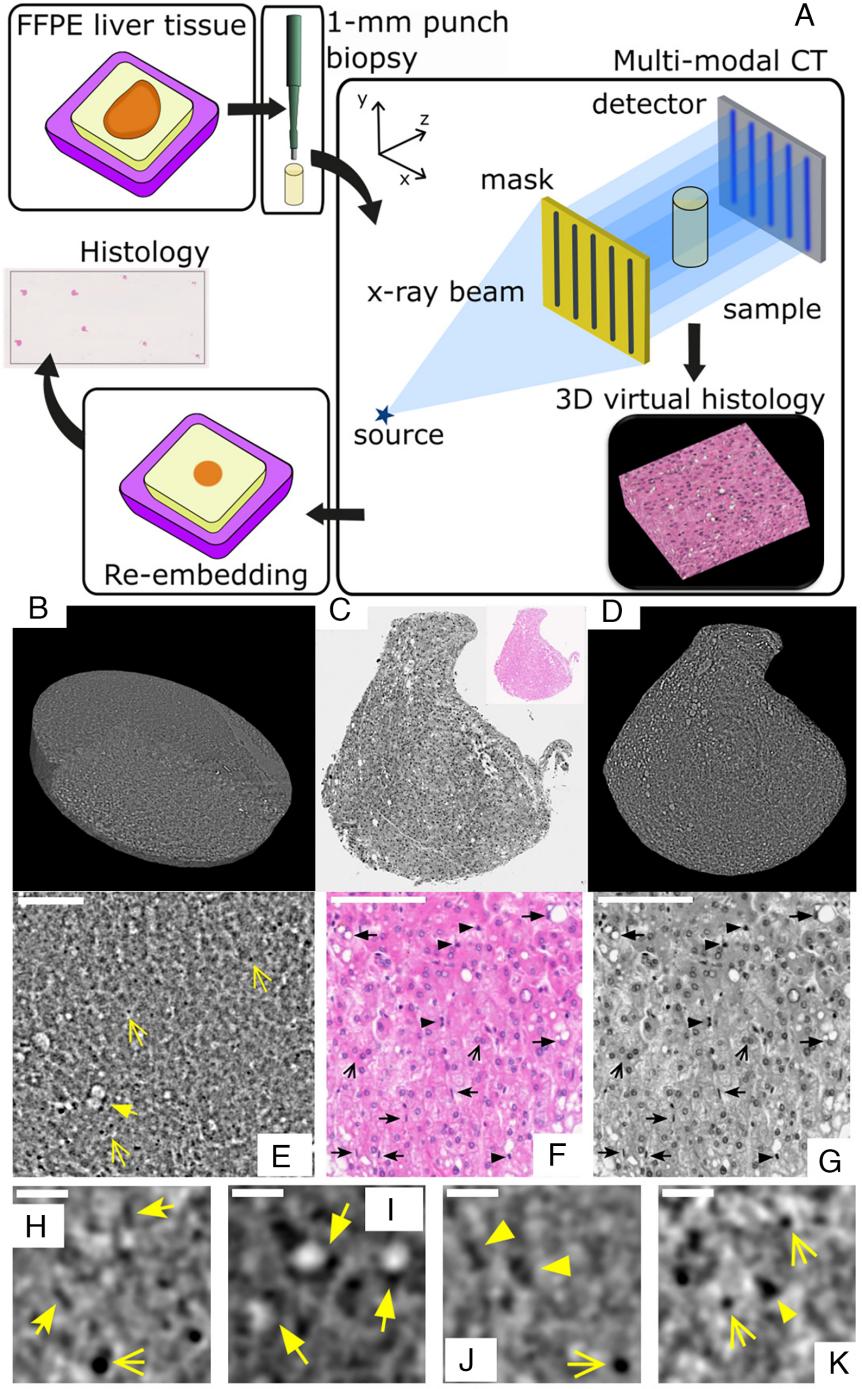
Three-dimensional imaging of the liver biopsy punch. (*A*) Schematic representation of the experimental workflow and diagram showing the imaging technique used in this work. (*B*) Volumetric representation of the electron density data. Sample diameter 1 mm. (*C*) An H&E histological slide converted to grayscale for ease of comparison alongside the original color image in the *Inset*. (*D*) A virtual slice of the electron density volume chosen at a similar orientation to the histology. Region of interest in the CT data (*E*) and in H&E histology shown both in color (*F*) and gray (*G*) scale. (Scale bars, 100 μm.) (*H*–*K*) ROIs extracted from the CT data (100 × 100 μm^2^). (Scale bar, 20 μm.) Different arrow styles are used to highlight different histological features in panels (*E*–*J*). Open arrows are used to identify hepatocytes nuclei, notched arrows for endothelial cells nuclei, filled arrows for vesicular fat metamorphosis and arrowheads for lymphocytes nuclei. The color scale of the electron density maps is inverted (darker = denser) in analogy with histology, to obtain darker nuclei on a brighter background.

A 1-mm-diameter biopsy punch was extracted from FFPE liver tissue and imaged with the multimodal X-ray microscope detailed in *Materials and Methods*. The design of the experimental workflow and a schematic representation of the imaging method are depicted in Fig. 1*A*. Fig. 1 shows a three-dimensional rendering (*B*) and a virtual slice (*D*) of the electron density map of the punch biopsy. The orientation of the virtual slice was selected to closely match the orientation of an adjacent H&E slide obtained from the same sample (*C*). In addition to the original color scale version [*Inset* of panel (*C*)], histology was converted to grayscale for ease of comparison with the phase CT slice. Smaller Regions of Interest (ROI) from virtual and H&E slides (Fig. 1 *E* and *F*, respectively) reveal comparable features, including cell nuclei (hepatocytes, indicated by open arrows) and vesicular fat metamorphosis (filled arrows). The latter (also known as fatty change) is a pathological condition of the liver that, at the microscopic level, presents with the deposition of fat in the hepatocyte cytoplasm and the displacement of the hepatocyte nucleus at the periphery of the cell. ROIs for the virtual histology (Fig. 1*G*–*J*) demonstrate the possibility to differentiate cell types based on their nuclear morphology. In addition to hepatocytes (open arrows), endothelial cells nuclei (notched arrows) and lymphocytes (arrowheads) are identified. For reference, the corresponding cell types are labeled in the histology of panel (*F*). For comparison, the same sample was also imaged using two commercial micro-CT scanners, and a comparative analysis is reported in *SI Appendix*. Note that the color scale of the electron density maps, here and elsewhere in the manuscript, was inverted for ease of comparison with histology, where nuclei appear darker on a brighter background. We also note that only an approximate registration was possible between phase CT and histological slices. This limitation arose from the presence of cracks within the sample, which introduced artifacts in some CT slices made it impossible to determine the precise cutting angle of the corresponding histological section.

### Segmentation and Nuclear Morphology.

**Fig. 2.**
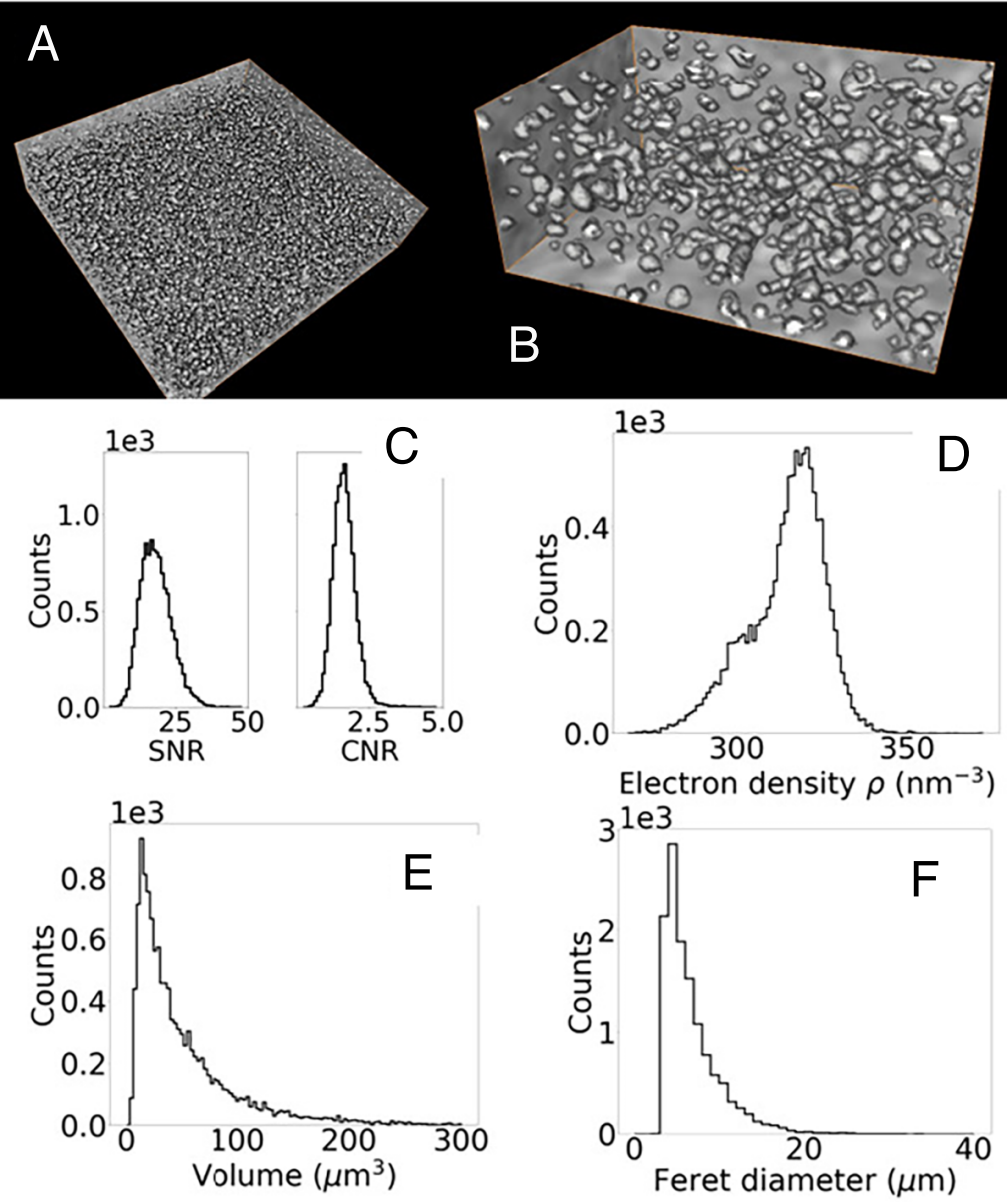
Automatic segmentation and nuclear morphology. (*A*) Three-dimensional rendering of segmented nuclei and a zoomed-in region (*B*). (*C*) SNR and CNR distributions calculated for all segmented nuclei. Characterization of nuclear morphology: electron density distribution (*D*), volume (*E*), and maximum Feret diameter (*F*).

Using the automatic segmentation algorithm detailed in *Materials and Methods*, cell nuclei were segmented in the electron density CT data. Three-dimensional visualizations of segmented nuclei are shown in Fig. 2 *A* and *B* at different magnification. For each of the segmented nuclei, Signal-to-Noise ratio (SNR) and Contrast-to-Noise ratio (CNR) were calculated, comparing the signal measured in the nucleus and in the immediate surroundings (Fig. 2*C*). These figures of merit were calculated as $SNR=S_{i}/\sigma_{B,i}$, and $CNR=S_{i}-B_{i}/\sigma_{B,i}$, with $\sigma_{B,i}$ being the SD of the background signal of the *i-th* nucleus ($B_{i}$) and $S_{i}$ its signal. A mean SNR = 18.33 ± 0.05 and a mean CNR = 1.673 ± 0.003 suggests that nuclei can be easily detected above the background. The microscope used in this work allows for the quantitative retrieval of the phase signal, proportional to the electron density (see *Materials and Methods* and *SI Appendix*). The electron density measured for segmented nuclei is reported in Fig. 2*D*. The mean electron density measured for nuclei in liver tissue was ρ=314 nm^−3^, with SD $\sigma_{\rho }=11$ nm^−3^. This value is consistent with values reported in literature (13), albeit for a different tissue type. Measured parameters associated with nuclear morphology are shown in Fig. 2, including nuclear volume (e) and maximum Feret diameter (f). The mean volume for the segmented nuclei was measured to be $\hat{V}=54\;\mu$m^3^ with a SD of $\sigma_{\hat{V}}=62\;\mu$m^3^, suggesting a strong heterogeneity in the nuclear size. The mean value of the three-dimensional Feret diameter was $\hat{d}=6.7\;\mu$m, characterized by a similarly broad distribution, resulting in a SD of $\sigma_{\hat{d}}=3.5\;\mu$m. Given that histology is inherently a two-dimensional technique while CT provides three-dimensional information, direct comparison of nuclear morphological properties between the two modalities is neither straightforward nor necessarily accurate. Nevertheless, the Feret diameter of nuclei measured in histological sections (*Materials and Methods*) yielded in a mean value of $\hat{d_{\mathrm{histo}}}=7.0\;\mu$m, which is comparable with the results obtained from our electron density CT. Additional morphological parameters, such as major (minor) axis length and eccentricity, are reported in *SI Appendix*.

### Virtual Histology.

**Fig. 3.**
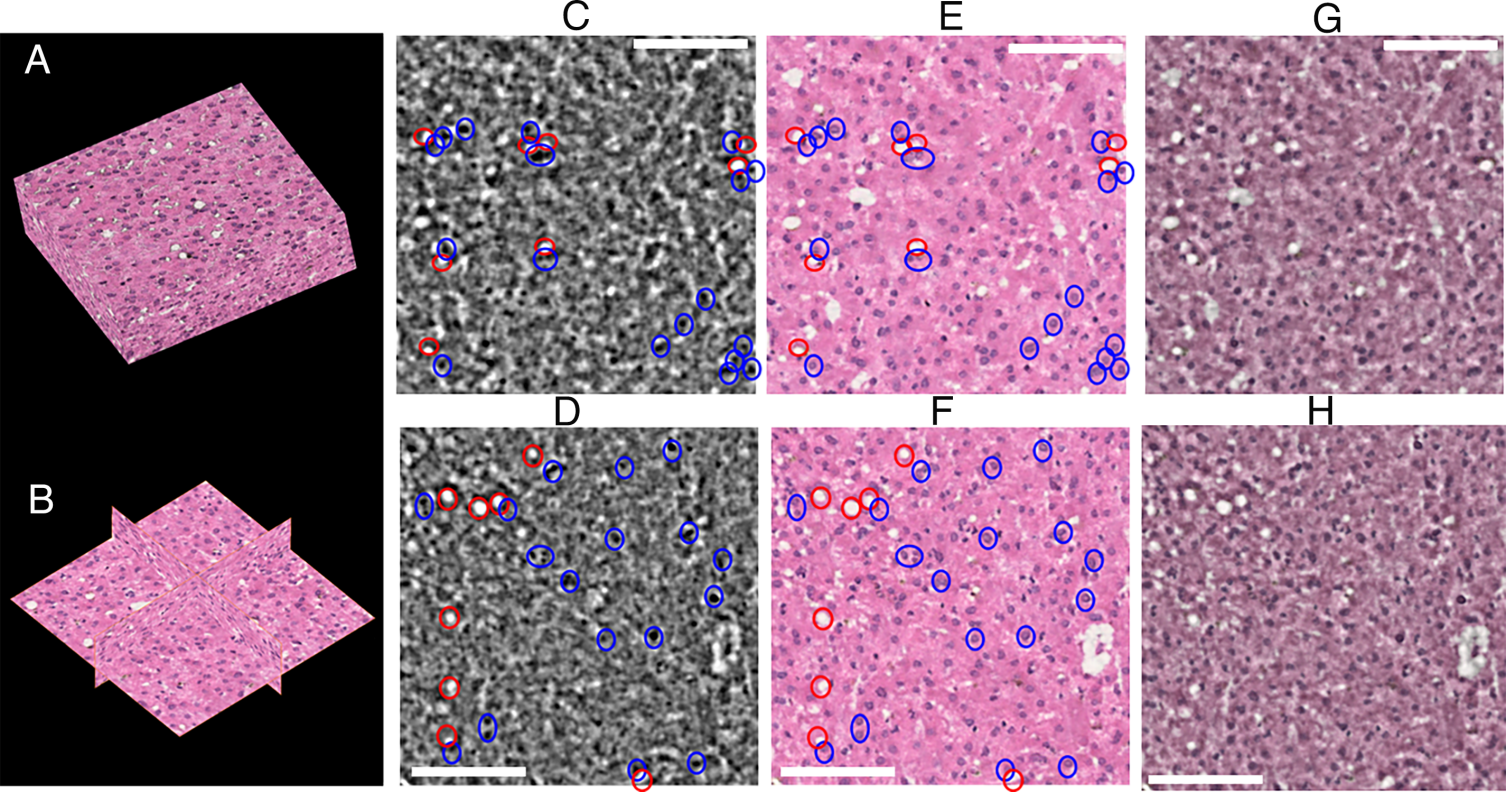
Three-dimensional virtual histology generated from the phase CT data. Panels (*A* and *B*) show a three-dimensional rendering of the generated histology data and cross-sectional views, respectively, highlighting the volumetric nature of the generated dataset. 2D slices (318 × 318 μm^2^) are shown in panels (*C* and *D*) with correspondingly generated H&E histology (*E* and *F*). Paired generated histology and phase slices were annotated by a paediatric pathologist (JCH) with fat vesicles shown in red and nuclei in blue. Additional annotated slices are included in the *SI Appendix*. Each two pairs of images are overlaid and shown in panels (*G* and *H*) (50% transparency). Scale bars 100 μm.

While a wealth of quantitative information at cellular scale can be extracted from the dataset of Figs. 1 and 2, direct interpretation of micro-CT images may be challenging for clinicians and biomedical researchers accustomed to conventional histological images. Furthermore, computational workflows designed for histological datasets may not be readily applicable to electron density maps. To address this limitation, we used a Machine Learning (ML)-based approach to convert electron density maps into a representation resembling H&E-stained histology. The style transfer was implemented using a Generative Adversarial Network (GAN), a class of models known for producing highly realistic synthetic images (40, 41) (*SI Appendix*). Fig. 3 illustrates the outcome of the style transfer, with panels (*A* and *B*) highlighting the volumetric nature of the generated data. A two-dimensional comparison is also provided (Fig. 3*Insets*, *C*–*H*), showing paired images from both domains and an overlaid image (50% transparency). Key liver features, such as hepatocyte nuclei and vesicular fat, are faithfully reproduced in the transformed images, demonstrating strong structural consistency between the modalities. To further corroborate this claim, paired CT and virtual histology slices were annotated by a pediatric pathologist and fellow of the Royal Society of Pathology (JCH). The pathologist marked fat vesicles in red and nuclei in blue. Full annotation of the images was not pursued because the high density of nuclei would have rendered the annotations unclear by making the images excessively visually overloaded. Additional annotated paired slices are provided in *SI Appendix*.

### High-Content Imaging.

**Fig. 4.**
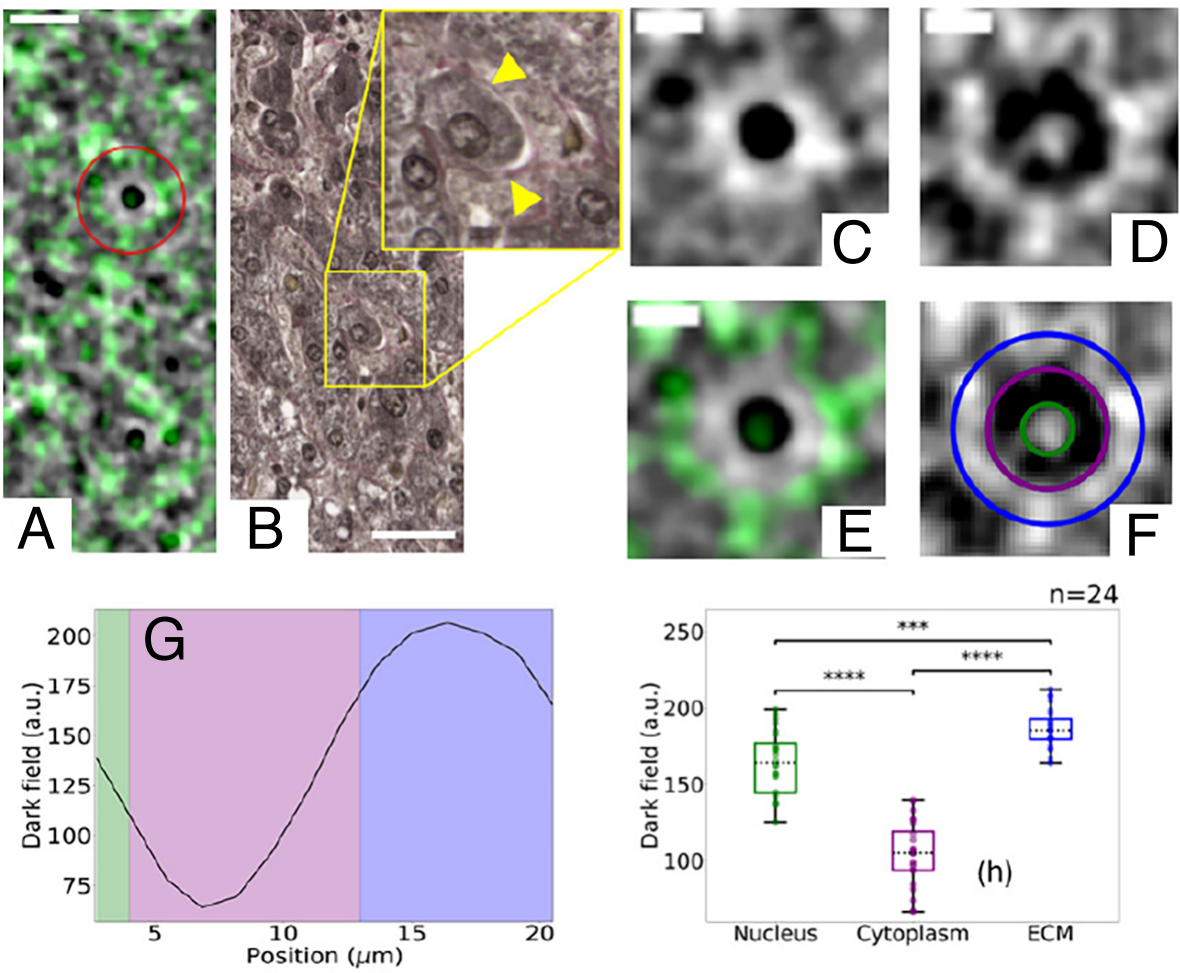
High-content histology: virtual staining using the dark field contrast channel. (*A*) High-content image of a ROI from the liver sample, with the phase signal displayed in grayscale and the dark field signal overlaid in green. (Scale bar, 25 μm.) (*B*) Trichome staining of a histological section from the same sample. A magnified view of a cell is shown in the zoom-in panel. Collagen in the extracellular matrix (ECM) appears pink and is marked with yellow arrowheads. (Scale bar, 25 μm.) (*C*–*E*) Zoom-in on an individual cell marked by the red circle in panel (*A*), displaying electron density map (*C*), dark field map (*D*), high-content image with phase in grayscale and dark field in greens (*E*). Three different regions can be identified in the dark field image, highlighted by green, purple, and blue annuli in (*F*). [Scale bars in (*C*–*F*) are 10 μm.] (*G*) Radial profile of the dark field signal in panel (*F*), with regions corresponding to the nucleus, cytoplasm, and ECM shaded in green, purple, and blue, respectively. (*G*) Values of the dark field signal associated with nucleus, cytoplasm, and ECM for individual cells (n=24). Boxplot elements: central mark indicates the median, and the *Bottom* and *Top* edges of the box indicate the 25th and 75th percentiles, respectively. The whiskers extend to the most extreme data points, not considered outliers. The statistical comparison was performed by the Wilcoxon rank sum test (two-sided). A *P*-value ≤ 0.05 is considered as statistically significant. We indicate P≤0.001 as (∗∗∗) and P≤0.0001 as (∗∗∗∗).

We demonstrate that the combination of complementary contrast channels, as provided by the multimodal X-ray microscope (described in detail in *Materials and Methods*), enables high-content imaging spanning both micro- and nanoscale length scales. Fig. 4*A* shows a representative region of interest (ROI) from the sample, where phase contrast is rendered in grayscale and dark field contrast in shades of green. The two contrast channels reveal different structural features within the image. With a real resolution limit (as opposed to voxel size) of 1 μm, the phase signal quantifies the electron density of sample features within the system’s spatial resolution, while the dark field channel highlights ensembles of subresolution features (i.e., at the nanoscale) (39). A magnified region of the high-content image (Fig. 4*C*–*E*) shows an individual cell, where the dark field signal is pronounced both inside the nucleus and at the cell periphery.

Fig. 4*G* presents a radial profile of the dark field signal for the cell shown in panel (*D*), centered on the nucleus. Two distinct peaks are observed. The first corresponds to the nucleus, as also evident in the electron density map [panel (*C*)]. The second peak appears at the cell periphery, separated from the nuclear signal by a trough corresponding to the cytoplasmic region, which exhibits lower dark field intensity. An enhanced dark field signal associated with the nucleus has previously been reported (42), attributed to X-ray scattering from densely packed nuclear components. Similarly, the low signal in the cytoplasm is consistent with low density of scattering structures on the nanometric scale. The second peak at the periphery is likely associated with collagen in the extracellular matrix (ECM), known to produce strong X-ray scattering due to its microscopically heterogeneous structure (39, 43). The three regions—nucleus, cytoplasm, and ECM—are shaded green, purple, and blue, respectively, in the radial profile [panel (*G*)] and are annotated with correspondingly colored annuli in the dark field image [panel (*F*)]. A histological section from the same tissue, stained with trichrome (a collagen-specific stain), is shown in Fig. 4*B*, where collagen adjacent to the cell membrane is highlighted by yellow arrowheads.

Finally, Fig. 4*H* presents a boxplot of the dark field signal measured across 24 individual cells, for each of the three identified regions. A two-sided Wilcoxon rank sum test was performed to assess statistical differences in the dark field signal measured in the three different regions, with *P*-value ≤ 0.05 considered to be statistically significant. The analysis revealed statistically significant differences in signal intensity between the three regions (P≤0.001 and P≤0.0001), further supporting the ability of the dark field channel to observe nanoscale structural domains.

## Discussion

The first key result presented in this article is the demonstration that multimodal X-ray microscopy enables three-dimensional histology with subcellular resolution (Fig. 1), offering a viable alternative to conventional histology in bridging the gap between macroscale and nanoscale imaging of soft tissues. Conventional histology, making use of specialist stains (with H&E being the most common choice) produces intrinsically segmented images. In a H&E histology slide, nuclei appear in purple/blue and cytoplasm and extracellular matrix in pink. Hence, it is key for the successful translation of the technology presented in this work to include a robust segmentation stage. The spatial resolution and image contrast achieved with the X-ray microscope allow for the detection of nuclei with a high SNR (Fig. 1*C*), well above the Rose criterion of SNR = 5. This enabled the straightforward implementation of an automatic segmentation workflow (*SI Appendix*). The simplicity and speed of the algorithm, combined with the lack of need for external inputs (e.g. seeding, training datasets), makes it possible to envision automatic scanning and segmentation workflows facilitating high-throughput imaging.

Leveraging on the reliable segmentation of cells’ nuclei and the availability of ML algorithms for style transfer, we demonstrated three-dimensional X-ray virtual histology with H&E staining. The resulting datasets are fully compatible with existing histopathological analysis tools and are immediately interpretable by clinicians and life scientists trained in traditional histology. This result represents a key advancement for volumetric tissue imaging. In fact, conventional histology can lead to misinterpretation of three-dimensional structure as a result of distortions from processing and sectioning (44). While three-dimensional reconstruction of multiple serial slides is possible, this is often problematic as it leads to the exhaustion of limited available tissue. Additionally, the reliability of the reconstructed volumes is often limited, as biases can be introduced in the reconstruction process (1). Although conventional histology still offers a superior spatial resolution (<1 μm) and enables molecular staining, here we demonstrated that the method proposed in this paper can overcome these limitations, by providing inherently three-dimensional H&E volumes in a nondestructive fashion, and so preserving tissue for ancillary investigations.

Another core strength of this approach is the quantitative nature of the intensity-modulation phase-contrast technique enabled by the use of quasi-monochromatic radiation, which provides direct measurement of electron density. Unlike propagation-based techniques (33), which rely on restrictive assumptions on sample homogeneity and chemical composition to obtain an estimate of electron density (37), the proposed method measures electron density directly, with the added advantage of a potential lower uncertainty and is less prone to systematic errors. By using nanoscale resolution imaging techniques at specialized synchrotron facilities, Bhartiya et al. (45) demonstrated that quantifying nuclear electron density can provide crucial information on the nuclei’s genetic components and give information on cells’ health and on chromosomal aberrations. While beyond the scope of our present work, our results pave the way to achieve this in the standard laboratory environment, combining cell type identification using nuclear morphology (e.g. volume, Feret diameter, eccentricity, see Fig. 1*F*–*I* and *SI Appendix*) with quantification of electron density.

The second major innovation of this work is the possibility to produce stain-free high-content imaging, combining complementary contrast channels. The use of different histological stains is common practice in histology, as it allows for identifying different functional structures, e.g. in this work we showed H&E and trichrome stains to highlight nuclei and collagen in the ECM (Figs. 1 and 4). A main limitation associated with the use of multiple stains, beyond cost and time requirements, is that the process of obtaining histological slides is destructive, resulting in the impossibility of running comparative analysis on the same tissue slice. Here, we showed that combining electron density and dark field maps allowed the stain-free identification of nuclei and ECM, making this method a high-content imaging modality.

The X-ray microscope developed in this work is a precommercial prototype designed for imaging tissue volumes on the order of 1-mm^3^. Nevertheless, its design allows for scalability to larger volumes, enabled by its aperture-driven resolution (46), which arises from the use of intensity-modulation masks. In contrast, propagation-based systems aimed at virtual histology with cellular resolution typically have their spatial resolution constrained by the chosen detector pixel size and source focal spot size. For this reason, nano-focal sources with high geometrical magnification (26, 27) or microfocal sources with optical magnification (29, 33) were used. Since spatial resolution is intrinsically linked to the source and detector characteristics, scaling to accommodate larger sample sizes remains a significant challenge. This often results in reduced resolution when imaging at larger fields of view (FoVs). In the prototype developed here, however, spatial resolution is decoupled from these parameters, allowing for imaging of larger FoVs without sacrificing resolution. While scaling up the FOV without compromising resolution represents a significant advancement in biomedical imaging, it does introduce certain trade-offs. The intensity-modulation masks that provide the aperture-driven resolution necessitate sample dithering, i.e., stepping the sample perpendicularly to the apertures to ensure full illumination. They also reduce the available flux at each dithering step; however, the total number of photons, and therefore the quantum component of the noise profile of each dithered image, remain unchanged due to the dithering process. The dithering process, however, results in longer scan times: a limitation that is partially mitigated by employing a cycloidal scanning approach (47) (*SI Appendix*). In addition, the intensity-modulation masks themselves constitute an additional specialized component that must be integrated into and aligned within the imaging setup.

## Materials and Methods

### Liver Tissue.

Formalin-Fixed and Paraffin-Embedded (FFPE) liver tissue, mounted on a conventional histology cassette, was obtained from the Department of Pathology at the Memorial Sloan Kettering Center.^*^ A 1-mm diameter biopsy punch was obtained from the paraffin block and imaged with the X-ray microscope. See Fig. 1*A* for a schematic representation of the experimental workflow. The biopsy core was then re-embedded in paraffin, sectioned in 4 μm-thick slices, stained with hematoxylin and eosin (H&E), and then scanned with a Whole Slide Imaging Scanner (WSI) system (Nanozoomer S60, Hamamatsu Photonics, Hamamatsu, Japan) at 40× magnification (0.23 μm/pixel). An additional slice was stained with Trichrome and scanned using the same WSI equipment. It should be noted that the obtained histological images show a ≈10% increase in the longitudinal direction compared to CT, due to shear deformations as a result of the slicing process. Nuclei in the H&E histology images were segmented and their size quantified using the function Cell detection of the commercially available software QuPath (v. 0.5.1) (48).

### X-Ray Microscope.

The X-ray microscope used in this work consists of a rotating anode Cu source (Rigaku) with a focal spot size of 70 μm. The X-ray beam passes through a flat multilayer monochromator (49) selecting the Cu K_*α*_ lines (≈8 keV). An intensity-modulation mask is placed at 70 cm from the focal spot. The mask, detailed in ref. 50, is a 10-μm-thick Au membrane featuring 1 μm wide apertures on a 7.5 μm period. The sample, whose position is adjusted with six degrees of freedom, is placed in close proximity to the mask. A propagation distance of 35 mm is set between sample and detector. The detector (Rigaku, XSight™ Micron LC X-ray sCMOS), based on an indirect detection approach, features an effective pixel size of 0.65 μm.

### Intensity-Modulation Phase-Contrast Imaging.

The X-ray microscope is based on the use of an intensity-modulation mask to detect phase shifts. The mask shapes a broad X-ray beam into an array of beamlets so that changes in intensity, position, and width of each beamlet can be quantified and associated to the sample’s properties (51). A schematic diagram of the intensity-modulation approach is shown in Fig. 1*A*. Assuming a bell-shaped distribution for the shaped beamlets, they can be individually characterized in terms of amplitude A, central position μ and variance $\sigma^{2}$. Measuring these parameters with {A,μ,σ} and without $\left\{A_{0},\mu_{0},\sigma_{0}\right\}$ sample at each angular position θ of a tomographic scan, allows the retrieval of transmission L, refraction R, and dark field DF. For a monochromatic X-ray beam and following ref. 52, these can be written as

[1] $$\begin{array}{c} L\left(x_{a},\theta ;\;\lambda \right)=-ln\left(\frac{A}{A_{0}}\right)=\int_{O}\mu \left(x_{a},\theta ,z;\;\lambda \right)dz, \end{array}$$

[2] $$\begin{array}{c} R\left(x_{a},\theta ;\;\lambda \right)=\frac{\mu -\mu_{0}}{\Delta z}=\int_{O}\frac{\partial }{\partial x}\delta \left(x_{a},\theta ,z;\;\lambda \right)dz, \end{array}$$

[3] $$\begin{array}{c} DF\left(x_{a},\theta ;\;\lambda \right)=\frac{\sigma^{2}-\sigma_{0}^{2}}{\Delta z^{2}}=\int_{O}\sigma_{f}^{2}\left(x_{a},\theta ,z;\;\lambda \right)dz, \end{array}$$

with Δz being the propagation distance between sample and detector along the optical axis z, $x_{a}$ the location of a specific mask aperture (and hence beamlet), λ the wavelength of the monochromatic beam, O the extent of the sample along the optical axis, and μ=(4π/λ)β the linear attenuation coefficient. β and δ are the imaginary part and the decrement from unity of the real part of the complex refractive index, respectively. $\sigma_{f}^{2}$ is the variance of the scattering distribution function ϕ. Eqs. **1**–**3** can be inverted by tomographic reconstruction to obtain quantitative three-dimensional maps of μ, δ and $\sigma_{f}^{2}$. Of particular interest for this work is the phase signal δ, as it allows obtaining maps of δ that can be directly and quantitatively linked to the electron density ρ by

[4] $$\begin{array}{c} \delta \left(\lambda \right)=\frac{2\pi r_{e}\rho }{k^{2}} \end{array}$$

with $r_{e}$ the classical electron radius and k=2π/λ the wave number. The quantitativeness of the retrieved electron density is discussed in *SI Appendix*.

### Imaging, Retrieval, and Reconstruction.

The biopsy punch was imaged using the X-ray microscope. Projection images were obtained over 360^°^ in steps of 0.25^°^. At each angular position, the sample was dithered, i.e. stepped in subaperture steps (0.75 μm) for a total shift equal to the mask period (7.5 μm) to deliver full sample illumination (53). An exposure time of 30 s per frame was used. The total live time for this experiment was 120 h and real time 125.4 h, with a dead time corresponding to 4% the real time. Options for decreasing the number of required dithering steps are discussed in *SI Appendix*, showing the potential of reducing the scan time by at least 50%. Hardware modifications, including the use of a higher power source and higher efficiency detector could further reduce the scan time. Retrieval of transmission L, refraction R, and dark field DF was performed at each angular and dithering position using Eqs. **1**–**3**, and the resulting images for each dithering step interleaved, to yield images with resolution corresponding to the size of the mask apertures (1 μm). Retrieval of individual projections required between 5 and 8 min per image, depending on how quickly the fitting procedure converged. It should be noted that, although in this case the retrieval was performed after the experiment had been completed, it is also possible to carry out this data-processing task on the fly during data acquisition, since the retrieval of each projection is independent of all the others. Refraction images (Eq. **2**) were integrated along the direction of phase sensitivity (x) using the Hilbert transform. Each of the three independent tomographic datasets was reconstructed using the Core Imaging Library (CIL) (54, 55). Tomographic reconstructions were performed using a noniterative algorithm based on the Tikhonov regularization, with the gradient operator as regularization operator. The resulting optimization problem was solved using the Conjugate Gradient Least Square (CGLS) method. Phase integration and reconstruction required approximately 3 min per slice, and this task can be parallelized, as each slice is independent in a parallel-geometry imaging setup. Additional computations tasks, including segmentation and style transfer, are detailed in *SI Appendix*. Segmentation required approximately 9 min for a 382 × 355 × 90 μm^3^ volume. The style-transfer training phase took 45 min on an NVIDIA H100 for 200 epochs with a batch size of 32, while the conversion of electron-density maps to an H&E-stained volume required 48 s on an NVIDIA RTX A2000. All other computational tasks (i.e., retrieval, reconstruction, and segmentation) were performed on an Intel Xeon W-2265 (3.5 GHz, 12 physical cores) with 128 GB RAM.

## Supplementary Material

Appendix 01 (PDF)

Movie S1. Animation showing virtual slices through the reconstructed three-dimensional phase dataset. Within the volume, both cells’ nuclei and vesicular fat metamorphosis, a pathological state of the liver, are clearly identifiable. The same volume is then showed after style transfer to H&E histology, demonstrating the microscope capability to produce non-destructive three-dimensional virtual histology of unstained tissue.

Movie S2. An animation displaying axial slices of the liver sample’s region of interest (278×242 *μ*m^2^) imaged with the Nano3DX CT scanner with a pixel size of 0.63*μ*m, corresponding to the areas presented in Figure S5. A higher density of fat vesicles is visible, however, corresponding displaced nuclei are not observed.

## Data Availability

CT datasets and relevant code are available at https://doi.org/10.5522/04/31431553 (56).

## Footnotes

^*^The paraffin block was obtained from discarded deidentified tissue and, as such, exempt from ethical approval of Internal Review Board.
