# Supplementary material for: Three-dimensional high-content imaging of unstained soft tissue with subcellular resolution using a laboratory-based X-ray microscope
Source: Proc Natl Acad Sci U S A. 2026 Mar 17;123(12):e2525239123. doi: 10.1073/pnas.2525239123 (PMC13012051; doi:10.1073/pnas.2525239123)
Supplement: Supplementary file 1 — Appendix 01 (PDF) [file pnas.2525239123.sapp.pdf]

Supporting Information for

Three-dimensional high-content imaging of unstained soft tissue with subcellular resolution using a laboratory-based X-ray microscope

Michela Esposito, Alberto Astolfo, Yang Zhou, Ian Buchanan, Alexei Teplov, John Ciaran Hutchinson, Marco Endrizzi, Alexandra Egido Vinogradova, Olga Makarova, Ralu Divan, Cha-Mei Tang, Yukako Yagi, Peter D. Lee, Claire L. Walsh, Joseph D. Ferrara, Alessandro Olivo

Corresponding Author name.
E-mail: michela.esposito@ucl.ac.uk

This PDF file includes:

Supporting text
Figs. S1 to S10
Table S1
Legends for Movies S1 to S2
SI References

Other supporting materials for this manuscript include the following:

Movies S1 to S2

Supporting Information Text

Automatic segmentation of electron density maps

Electron density maps, obtained from the phase CT using Equations 2 and 4 of the main manuscript, were segmented to isolate individual cell nuclei. An example electron density image is shown in Figure S1 (a) with inverted colour scale to match grey scale histology (Figure 1 (c) of the main manuscript). Black and white top hat filters were applied to the CT datasets to identify nuclear and non-nuclear regions, respectively. The result of applying top hat filters to the electron density map is shown in Figure S1 (b). Top hat filters were used as labels for a random-walk-based segmentation algorithm (1). Figure S1 (c) shows the outcome of the random walk algorithm, with nuclei contours highlighted in green.

Segmentation labels were subsequently post-processed to cluster connected voxels, i.e. individual nuclei. The function *label* from the *measure* package in Scikit-Image (v. 0.22) library was used, using 1-connectivity. Properties of each labelled nucleus were then extracted using the function *regionprops* from the same library, including average intensity (i.e. electron density), volume, Feret diameter and the lengths of the major and minor axes. Eccentricity was calculated as the ratio of the latter two parameters. Labels were then used for calculating Signal-to-Noise and Contrast-to-Noise ratio (SNR and CNR) for individual nuclei. The mean signal $\bar{S}_i$ was calculated across all voxels assigned to each nucleus i. The mean background $\bar{B}_i$ was calculated as the mean voxel intensity within the bounding box of each segmented nucleus i, expanded by 2 voxels in each direction, while excluding pixels belonging to the nucleus itself. This process ensures that the signal in each nucleus is compared to the background in its immediate vicinity. SNR and CNR were calculate as follows: $SNR = S_i/\sigma_{B,i}$ and $CNR = S_i - B_i/\sigma_{B,i}$, with $\sigma_{B,i}$ being the standard deviation of the background signal of the i-th nucleus (B_i).

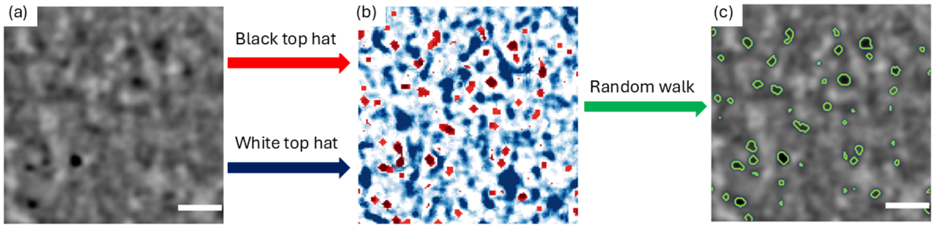

Fig. S1. Automatic segmentation workflow. (a) Phase image. Scale bar 20 μm. (b) Image obtained by applying white top hat (blue) and black top hat (red) filters to the image of panel (a). (b) is used as seed for a random walk algorithm leading to the segmentation of cells' nuclei. Contours of segmented nuclei are shown in panel (c). Scale bars 20 μm.

Validation of the segmentation workflow. In the main manuscript the segmentation workflow was applied to the largest available Region of Interest (ROI) that was clear of artefacts arising from air inclusions, with a volume of 382 $\times$ 355$\times$ 90 μm^3 (ROI 1). To validate the nuclear segmentation workflow, three additional artefact-free ROIs were identified and the segmentation workflow applied. The volumes of the additional ROIs are 150 $\times$ 150$\times$ 75 μm^3 (ROI 2) and 150 $\times$ 150$\times$ 110 μm^3 (ROIs 3 and 4). Electron density, major and minor axes length, Feret diameter, eccentricity and volume were quantified for all segmented nuclei, with results shown in Figure S2. A good agreement across the morphological parameters is visible across the four ROIs. Some differences, however, can be found for the electron density distributions. This might be due to anatomical or pathological variations within the specimens. Indeed, Eckermann et al. (2) demonstrated a statistically significant difference in the nuclear electron density between healthy and pathological brain tissue.

Quantitativeness of the electron density maps

As detailed in the Methods section, the X-ray microscope enables retrieval of the refractive index decrement, δ, from measured refraction data (see Equation 2), where $n = 1 - \delta + i\beta$ represents the complex refractive index. Furthermore, Equation 4 establishes that δ is directly proportional to the electron density, ρ. The quantitativeness of the measured δ maps is enabled by the quasi-monochromaticy of the X-ray source used. Here, we validate the quantitative accuracy of the electron density maps generated by our method. To do so, we performed tomographic imaging of two reference samples: a 150 μm diameter nylon wire and a 180 μm diameter polybutylene terephthalate (PBT) wire. A tomographic scan was conducted over a full 360° in increments of 0.5° increments, with the sample translated (dithered) in 2.5 μm steps. The refraction signal was retrieved, integrated, and used to reconstruct δ maps, which were subsequently converted into electron density values (ρ) using Equation 4. Figure S3(a) displays a reconstructed slice containing both test objects. Corresponding histograms of the measured ρ for two regions of interest (ROIs), marked by blue and red circles in panel (a), are shown in panel (b). The measured electron density for the nylon wire was ρ=290.6 nm^{-3}, with standard deviation σ=18.0 nm^{-3} and standard error s=0.3 nm^{-3}. The expected value, assuming the following chemical composition for the wire $(C_6H_{11}NO)_n$, is 280 nm^{-3}. The measured electron density for the PBT wire was ρ=334.1 nm^{-3}, with standard deviation σ=13.8 nm^{-3} and standard error s=0.3 nm^{-3}. The expected value, assuming the following chemical composition for the wire $(C_{12}H_{12}O_4)n$, is 312 nm^{-3}. Theoretical electron density values were obtained from tabulated δ values (3).

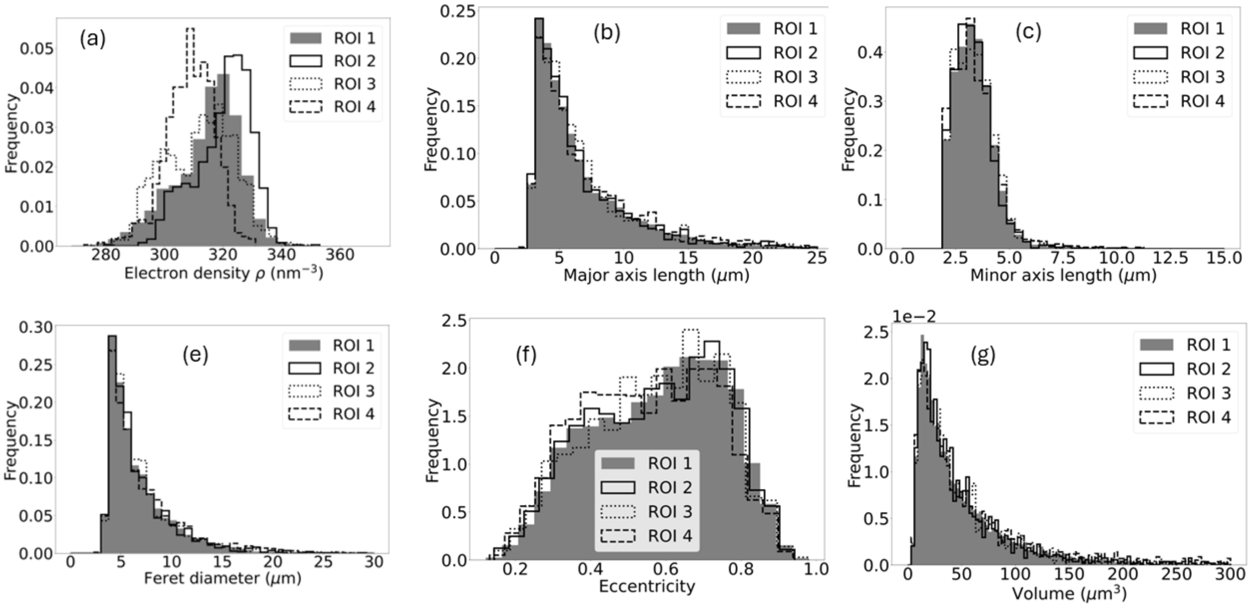

Fig. S2. Nuclear morphological parameters for different ROIs. Electron density (a), major (b) and minor (c) axes length, Feret diameter (d), eccentricity (e) and volume (f). ROI 1 (solid grey) corresponds to the dataset presented in the main manuscript.

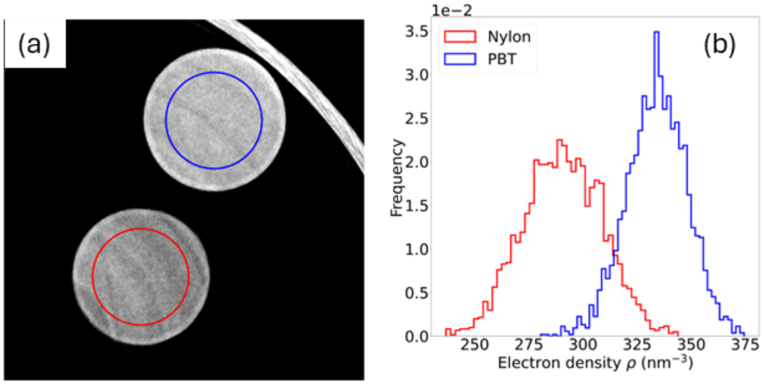

Fig. S3. a) Electron density map for nylon and a PBT wires and corresponding histograms for electron density (b).

The advantage of intensity-modulation phase-contrast microscopy: comparison with state-of-the-art micro-CT scanners

In the main article we discussed how the use of intensity-modulation masks allows for the system's spatial resolution to be determined by the mask aperture width (4), rather than by detector pixel pitch and focal spot size as it is the case for conventional imaging systems. For the microscope reported in this article, this leads to a *true* spatial resolution of 1 μm, corresponding to the capability of distinguishing two sample features placed 1 μm apart (5). Although it is common practice to quote the spatial resolution as the detector pixel size, in conventional imaging systems the true resolution is typically larger than the pixel size. Additionally, we have shown in the main manuscript that the combination of 1-μm spatial resolution and high phase sensitivity of our microscope allows for the detection of individual cellular nuclei in liver tissue.

To corroborate these claims, here we report on comparative data showing the potential of our microscope compared to state-of-the-art micro-CT scanners. To build this dataset, we scanned the same liver tissue sample presented in the main manuscript using two micro-CT systems. The first one was a 3DHistech custom-made micro-CT prototype, operating with a 2.7 μm effective pixel size, often used in clinical research to evaluate the whole resected tissue and whole tissue in FFPE block at MKSCC (6). The second system was a Rigaku Nano3DX micro-CT scanner. Since this features a Cu anode and a lower mean X-ray energy than the 3DHistech system, it is expected to provide higher soft-tissue contrast. The Nano3DX scanner uses a parallel-beam configuration and offers variable effective pixel sizes, achieved by selecting different objective magnifications at the detector. For this comparative study, we chose the smallest pixel size that currently would still allow the whole 1-mm diameter sample to be imaged,i.e. 0.63μm. Indeed, for most high-resolution scanners, a higher spatial resolution

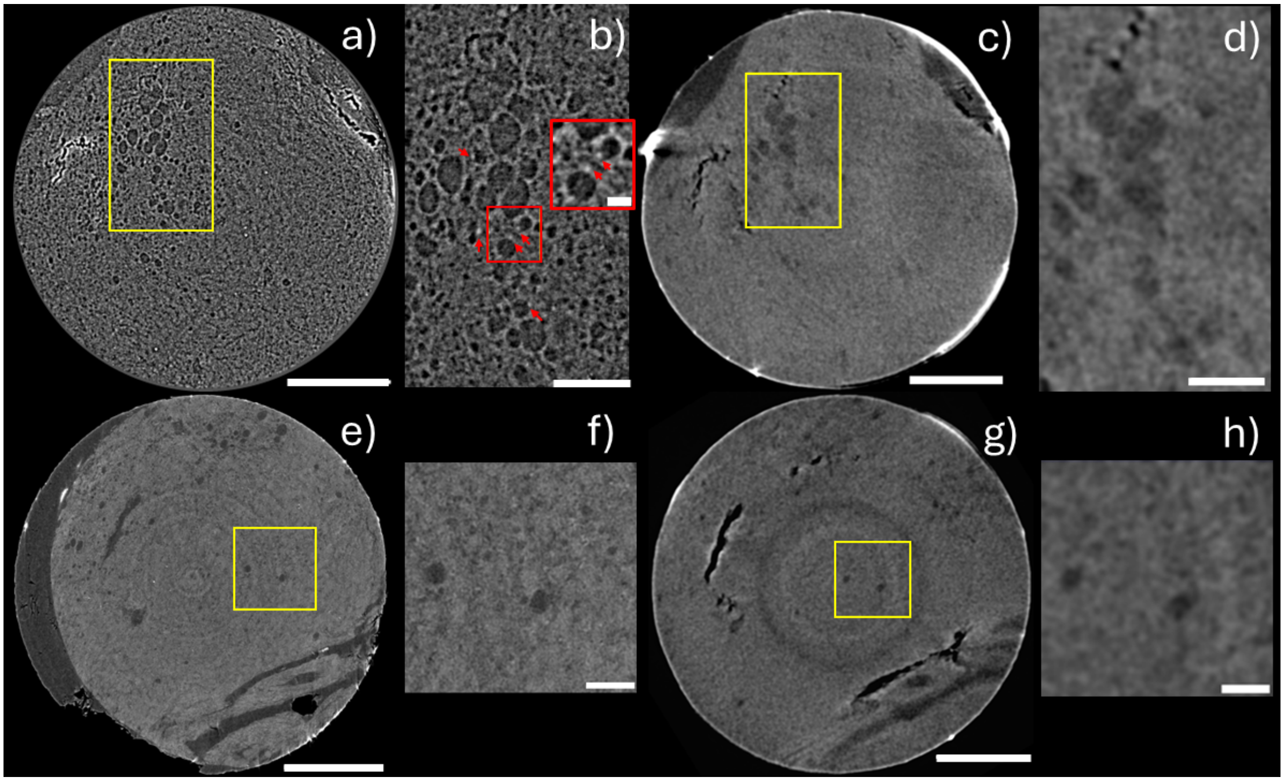

Fig. S4. Whole-slice comparison. Registered CT slices imaged with our intensity-modulation-based microscope (a) and with the 3DHistech prototype (c). ROIs, highlighted with yellow boxes, are shown in the corresponding magnified views in insets (b) and (d). Inset (b) is annotated with arrows indicating nuclei at the periphery of a fat vesicle. A magnified view of a smaller area of the sample (red box) shows two fat vesicles together with with corresponding displaced nuclei (20 μm scale bar). Registered slices imaged with the Nano3DX micro-CT scanner with 0.63 μm pixel size (e) and with the 3DHistech prototype one (g) are likewise shown, with ROIs magnified in insets (f) and (h). Scale bars in insets (a), (c), (e), and (g) are 300 μm. Scale bars in insets (b), (d), (f), and (h) are 50 μm.

comes at the expense of a reduced field of view, similarly to zooming in in optical microscopy. It is to be noted that the chosen pixel size is smaller than the expected spatial resolution of our microscope.

The liver tissue punch biopsy was first imaged with our microscope, followed by imaging on the 3DHistech prototype system. The sample was then re-embedded in paraffin wax, and several sections were taken for histological analysis, as described in the main article. From the remaining paraffin block, a new 1-mm core was extracted and subsequently imaged using the Nano3DX system.

Figure S4 shows a whole-slice comparison across the different imaging systems. Insets (a) and (c) show a registered liver tissue slice, imaged with our microscope and the 3DHistech prototype, respectively, with magnified views in insets (b) and (d) highlighting details of the liver fat vesicles. Due to subsequent sample processing (histology and additional punch biopsy), the same region was no longer available for imaging with the Nano3DX system; therefore, a slice at a different height in the original block is shown for this system. Insets (e) and (g) display registered slices for the 3DHistech prototype and Nano3DX systems, with magnified views of fat vesicles shown in insets (f) and (h). It should be noted that non-rigid deformation may have occurred during re-embedding and extraction of the new core, meaning that the registration between the two scans can only be considered approximate.

From Figure S4, it can be seen that contrast and resolution of the 3DHistech prototype system are insufficient to clearly resolve the microstructure of the liver fat vesicles, resulting in a loss of information for smaller feature sizes when compared with both our microscope and the Nano3DX system.

We then proceed to assess the capability of the Nano3DX micro-CT scanner to detect nuclei in liver tissue. To do this, we adopted the same approach used for cell detection with our microscope. Although nuclei can be found throughout the tissue, fat vesicles provide a particularly well-defined signature for their identification: fat vesicles appear as regions of lower density than the surrounding tissue with their associated nuclei displaced toward the vesicle periphery. An example of this can be seen in the magnified panel of Figure S4 (b). For this reason, we selected a region rich of fat vesicles and used this to identify nuclei. Figure S5 shows this region imaged with the Nano3DX system, where it can be seen that nuclei were not reliably detected at the periphery of fat vesicles. Supplementary Video 2 provides slice-by-slice visualizations of this volume and illustrates the absence of identifiable nuclei for most vesicles. In some instances, a higher-density structure near a fat vesicle can be seen and is highlighted by yellow arrows in Figure S5(c-d). Although these structures may represent candidate nuclei, their inconsistent appearance across different vesicles reduces the likelihood that they are true nuclei. Alternatively, even if these structures are indeed nuclei, their sparse occurrence across fat vesicles further supports the conclusion that the Nano3DX system did not

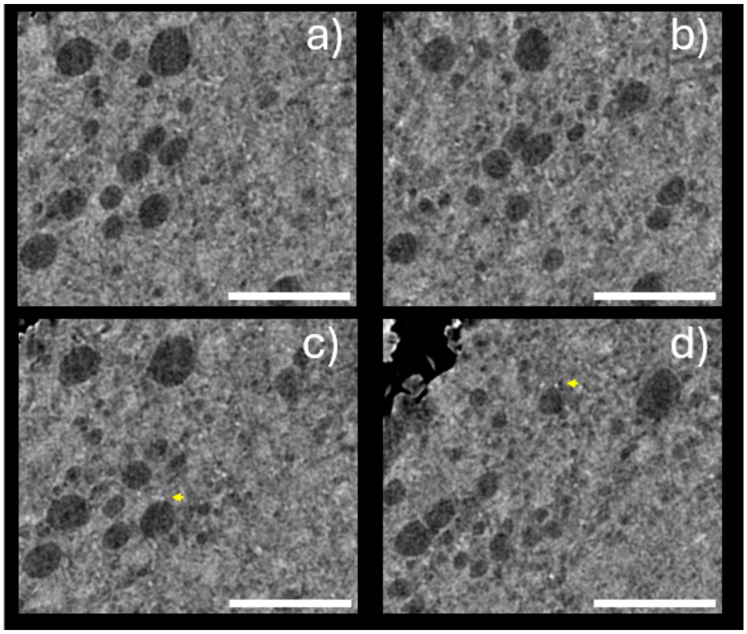

Fig. S5. (a-d) Slices showing liver areas rich of fat vesicles, imaged with the Nano3DX micro-CT scanner. Yellow arrows indicate possible candidate nuclei at the periphery of fat vesicles in insets (c-d). Scale bars are 100 μm.

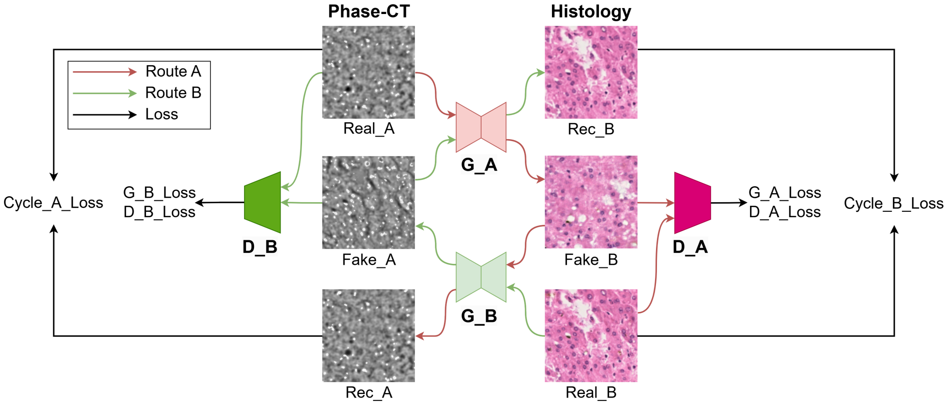

Fig. S6. A schematic representation of the cycleGAN architecture transferring style A (phase CT) to style B (H&E histology). The network comprises two generators (G_A, G_B) and two discriminators (D_A, D_B). Loss functions of individual components as well as over the cycles are indicated.

detect cell nuclei using a pixel size of 0.63 μm.

From this comparative analysis we can conclude the following: a) pathology-dedicated 3DHistech prototype with spatial resolution in the order of a few μm (2.7 μm pixel size) cannot accurately reproduce subcellular structures in liver tissue; and b) a high-contrast, low-energy micro-CT scanner (Nano3DX) did not detect nuclei in liver tissue using a pixel size of 0.63 μm. This latter result, obtained at a voxel size smaller than the resolution of our microscope, highlights the novelty of our proposed method and instrumentation when compared with state-of-the-art alternatives.

Virtual histology: converting electron density maps to H&E histology volumes

In Figure 3 of the main manuscript, we demonstrated how a machine learning (ML) approach based on a Generative Adversarial Network (GAN) enabled the translation of measured electron density maps into three-dimensional H&E-like volumes. Owing to the lack of paired data between the two domains, we employed a CycleGAN (7) architecture, an approach widely used in histopathology for stain transfer (8, 9). The network, designed to translate images from domain A (phase CT) to domain B

**Fold**	0	1	2	3	4
**Dice**	0.641	0.653	0.611	0.527	0.635

Table S1. Dice score measured on the test dataset for each fold training.

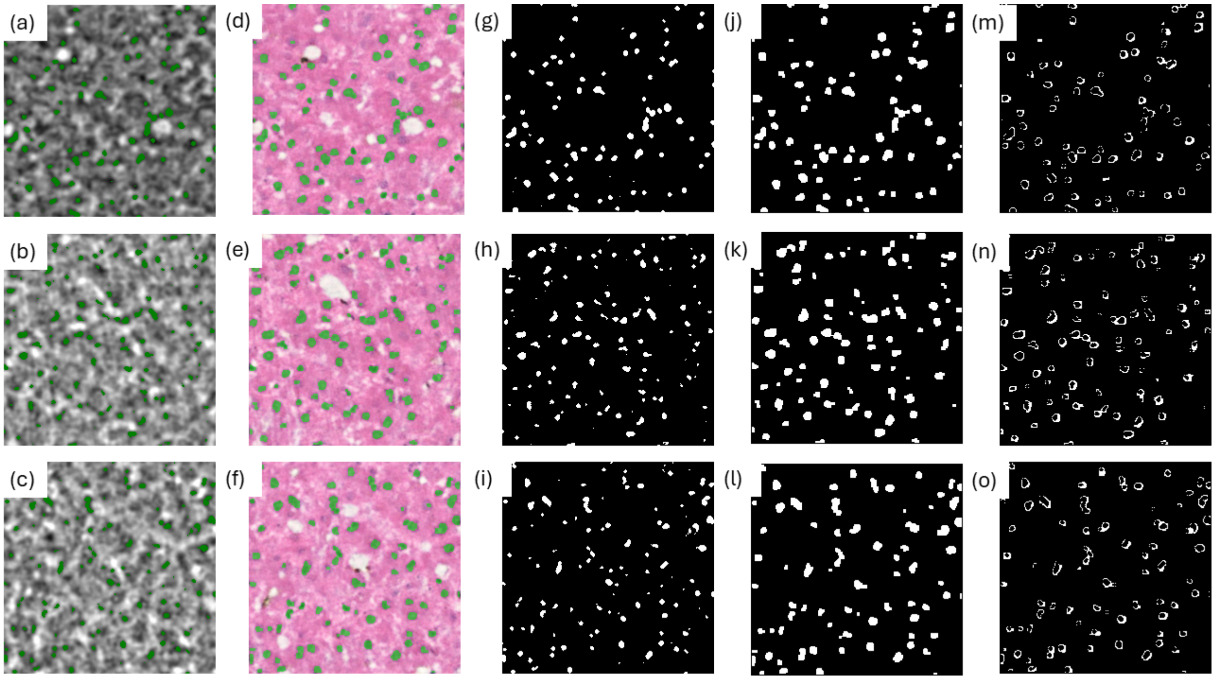

Fig. S7. Validation of the style transfer. Phase CT (a-c) and corresponding generated histology (d-f) with overlaid labels segmented nuclei (in green). Segmentation labels for CT (g-i) and generated images (j-l). Difference between generated and CT labels (m-o). All images are 168×168 μm^2.

(H&E histology), consists of two generators G_A (A→B) and G_B (B→A) along with two discriminators D_A and D_B. In the CycleGAN framework, the generators perform image translation between domains, while the discriminators aim to differentiate real images from those generated in each domain. The generators and discriminators are trained concurrently but independently. The training process involves several loss functions: the generative loss (G_x_Loss), the discriminative loss (D_x_Loss) and the cycle consistency loss (Cycle_x_Loss), where x denotes either domain A or B. The generative loss assesses the quality of image translation between domains, while the discriminative loss quantifies the ability of the discriminator to distinguish real from synthetic images. Through adversarial training, the model iteratively refines the generators to produce images that are increasingly realistic and challenging for the discriminators to classify. Additionally, the cycle consistency loss, proposed in (7), penalizes discrepancies between the original and reconstructed images, thereby reinforcing the fidelity of domain translation.

The training and testing datasets consisted of 1080 and 1322 patches for phase CT and histology, respectively. Histological images were obtained from the same sample, although not paired with the CT dataset. Each patch (224×224 pixels) was extracted by resampling the original data with a sliding window strategy and a stride of 100 pixels. Although data augmentation can improve the robustness of the training stage, it must be used cautiously as intensity changes, elastic deformations, and zero-padded rotations can disrupt imaging properties and cause domain-mapping failures. To avoid these issues, we trained directly on overlapping patches without augmentation. Phase CT images were pre-processed to artificially increase the contrast of the nuclei using the segmentation labels. A value corresponding to 80% of the maximum was assigned to pixels corresponding to segmented nuclei. For the histology data, the slide background was masked, i.e. areas of the histological slide not containing tissue were excluded. The he resampled patches included the background for less than 0.1% of the total pixels. The network was trained for 200 epochs. A learning rate of 0.0002 with a linear decay to 0 after 100 epochs was applied, to prevent over-fitting. The instance normalisation layer was used in the CycleGAN to normalise each patch individually before training. After completing the training of CycleGAN, only the generators (G_A and G_B) were utilized to perform image translation between the two domains, whereas the discriminators were excluded during the inference phase.

It should also be noted that the CycleGAN network used in this work was trained on histological images from the same sample, although these were not paired (i.e., registered) with the corresponding CT images. The model learns a style mapping between two domains: phase-contrast X-ray microscopy and H&E histology of liver tissues. When applied to other tissue types with substantially different morphological features, the current model is expected to show limited performance. To obtain reliable results in such cases, re-training or fine-tuning the network on new, tissue-specific data would be necessary.

To evaluate the performance of the CycleGAN model, we employed a 5-fold cross-validation strategy. An 80:20 split was applied to divide the data into training and validation datasets. For each fold, patches were randomly assigned either to the training or validation sets. The model was trained *ex-novo* and tested, with this process repeated five times (once for each fold) to ensure all data were used for validation. No paired samples were shared across training and validation sets to preserve domain independence. The same network architecture and training hyperparameters were used across all folds.

To quantitatively assess the correspondence between the generated H&E-like volumes and the reference CT images, we

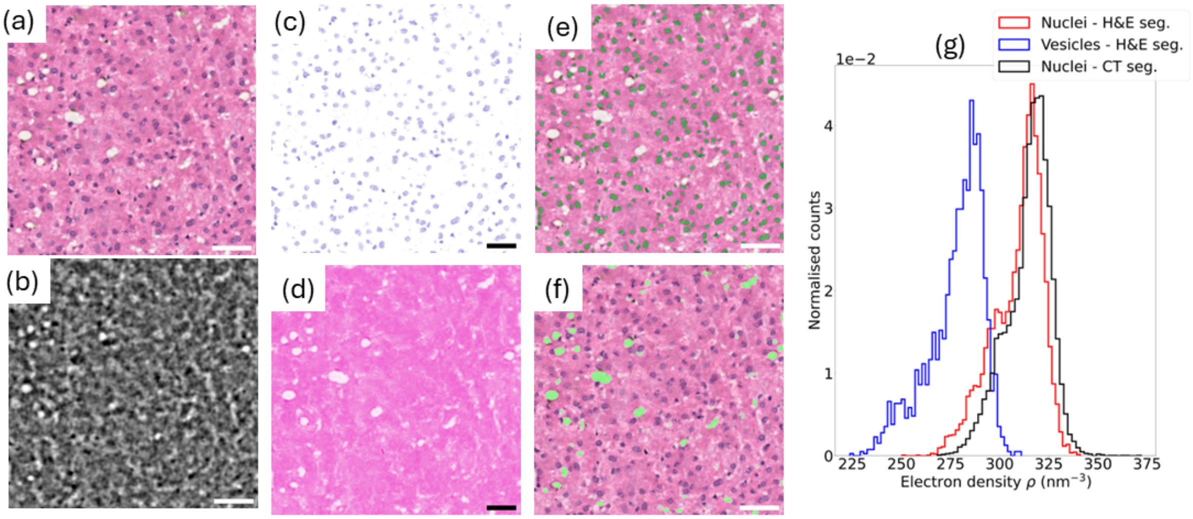

Fig. S8. Validation of the style transfer via electron density. A Virtual histology slice (a) and corresponding phase CT slice (b). Purple (c) and pink (d) channels resulting from colour deconvolution of image (a). These two images are the basis for the segmentation of nuclei and fat vesicles in the virtual histology slice. Highlighted in green are nuclei (e) and fat vesicles (f), overlaid with the original virtual histology image. Scale bars 50 μm. (g) Electron density distribution for the nuclei and fat vesicles calculated based on the labels resulting from the histology segmentation, compared with electron density of nuclei calculated from direct segmentation of the phase CT slices.

calculated the Dice Similarity Coefficient (DSC) on binary masks obtained from the validation datasets after training at each fold. The DSC quantifies the spatial overlap between predicted and reference regions, with values closer to 1 indicating higher agreement. DSC were computed on a per-slice basis and averaged across all validation samples. Binary masks were obtained by segmenting nuclei in both CT and generated histology images. DSCs obtained for each of the folds are reported in Table S1. Among them, fold 1 achieved the highest DSC of 0.653 and was therefore selected as representing the best correspondence between the two domains.

Figure S7 illustrates the model's ability to accurately reproduce nuclear structures in the generated histology. Panels (a–c) and (d–f) compare the original CT images and the synthetic histology, respectively, while panels (g–i) and (j–l) display the corresponding nuclear segmentations. The comparison shows that the positions and shapes of nuclei are generally well preserved from CT to histology; however, the nuclei in the generated images tend to appear enlarged. This is further highlighted in panels (m–o), which show the differences between the two sets of labels. These discrepancies are mostly localized around the nuclear boundaries and can be attributed to the difference in slice thickness between the histological sections (4 μm) used for training and the CT scans (0.75 μm). While this represents a key limitation of the virtual histology approach presented in this study, future work could mitigate this issue by using thinner histological sections—potentially as thin as 1-μm—which are expected to reduce such discrepancies.

To further validate the style-transfer approach from electron-density maps to H&E virtual histology, we assessed whether different morphological features in the generated H&E histology correspond to distinguishable electron density distributions in the phase CT dataset. Generated histological images were separates into two colour channels by making use of a colour deconvolution algorithm (10). An example of this process can be seen in Figure S8, showing a generated histology image (a), and the same image split into two of purple (c) and pink (d) colour channels. The purple channel allows for the identification of nuclei, while the pink channel facilitates the detection of fat vesicles, which appear as white circular structures in H&E histology. From the two colour channels, nuclei and fat vesicles were identified, using a simple intensity thresholding segmentation. Segmented nuclei and fat vesicles are shown in Figure S8(e-f). The segmentation labels were then applied to the corresponding phase CT images and electron density values for each of the identified structures evaluated. The electron density distribution for nuclei and fat vesicles, as identified from the generated histology, is shown in Figure S8(g). It can be seen that the two different electron density distributions correspond to the two distinct morphological features identified in the histology, with histology-segmented nuclei showing a higher density than fat vesicles, as expected. Additionally, the electron-density distribution for nuclei segmented directly in the phase-contrast CT data is also reported. A broad agreement is observed for the nuclei, although the distribution corresponding to CT-segmented nuclei exhibiting a higher mean value. This difference can be attributed to the overestimation of nuclear diameter in the generated histology, discussed in this section and shown in Figure S7, which results in a shift of the electron density distribution for histology-segmented nuclei toward lower values.

Opportunity for reducing exposure time: the cycloidal approach

The results presented in this work are achieved through the use of attenuation masks composed of alternated transmitting slits and absorbing septa, which produce a structured illumination pattern of intensity peaks and troughs (see Figure 1(a) of the main manuscript). As a result, at any given acquisition step, only a subset of the sample is probed. To ensure complete sample coverage, a *dithering* process is employed, whereby the sample is incrementally stepped perpendicular to the direction

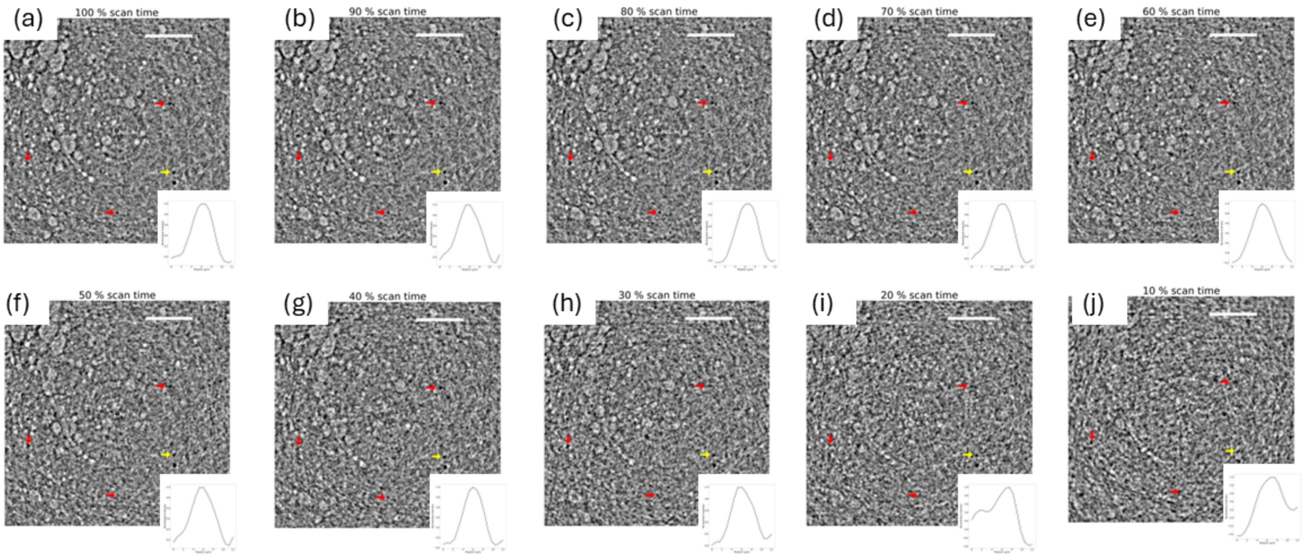

Fig. S9. Phase CT slice obtained using an increasing reduced number of dithering steps, leading to an exposure time from 100% of the original dataset (a) to 10% of it (j). Red arrows highlight a few reference nuclei. Yellow arrows identify a nucleus used for plotting its intensity profile, shown as an inset. Scale bars 100 μm.

of the transmitting apertures (see Figure 1 in the main manuscript), over a total distance equal to the mask's period. While guaranteeing phase sensitivity and the key advantage of aperture-driven resolution (5), this process entails stepping the sample at each acquisition angle of a tomogram, resulting in longer acquisition times.

To overcome this limitation, a cycloidal scanning approach has been proposed (11) where only a single step is acquired at each projection angle and the resulting sparse sinogram is interpolated. To evaluate the suitability of the cycloidal scan approach in the context of X-ray microscopy, the tomographic data of the liver tissue presented in the main manuscript have been undersampled according to the cycloidal scheme and reconstructed to simulate different scenarios of exposure time from 100% of the original dataset down to 10%. Specifically, sparse sinograms have been obtained from the original dataset by randomly sampling a number of dithering steps $N_d \in [1, 10[$ with $N_d = 10$ corresponding to the full dataset. The resulting sparse sinograms have been interpolated using a *Nearest-neighbour* interpolator, followed by phase integration and tomographic reconstruction, as detailed in the Methods section. Reconstructed tomographic slices are reported in Figure S9. Red arrows indicate specific features of interest, namely cell nuclei, while a yellow arrow marks a representative nucleus used for quantifying an intensity profile. The corresponding intensity profile is displayed as an inset in each panel. Although an increase in noise can be seen as exposure time decreases, even with a reduction in exposure time of up to 50% (i.e. $N_d = 5$, panel (f)), cell nuclei remain clearly resolvable, and no significant alterations are observed in the corresponding intensity profiles. However, for shorter scan durations, noise becomes the dominant factor and a decrease in resolution is observable, leading to a loss of resolution in high-frequency features such as nuclei. It is to be noted that all the tomograms in Figure S9 consist of the same number of angular projections, due to data availability. It has been shown (12) that for the cycloidal scheme to preserve aperture-driven resolution, specific sampling conditions, including angular, have to be met.

Additional annotated slices

Figure S10 shows additional paired CT and virtual histology slices annotated by a paediatric pathologist and fellow of the Royal Society of Pathology (JCH). Fat vesicles are highlighted in red and nuclei in blue. Full annotation of the images was not pursued because the high density of nuclei would have rendered the annotations unclear by making the images excessively visually overloaded.

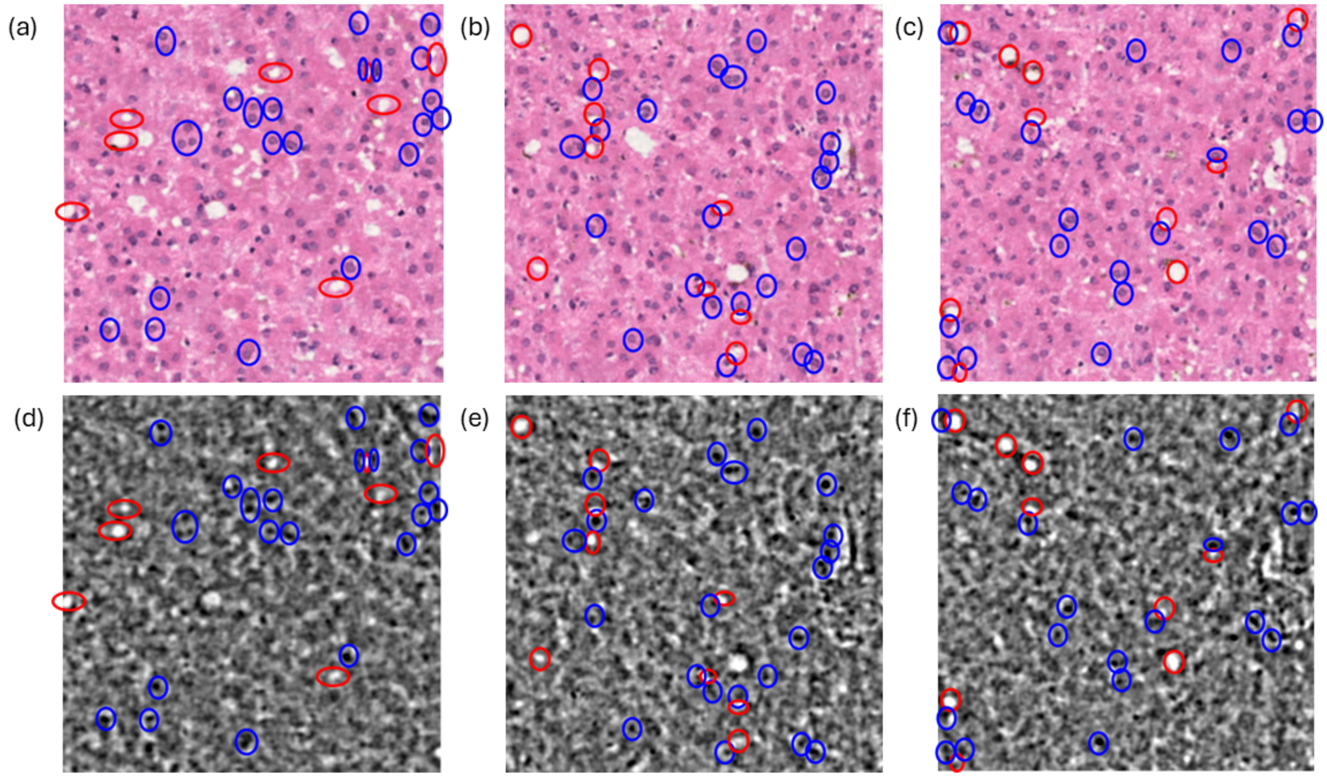

Fig. S10. Paired generated histology (a-c) and phase CT slices (d-f) annotated by a paediatric pathologist (JCH) with fat vesicles shown in red and nuclei in blue. The area of all the images is images is 318×318 μm^2.

Movie S1. Animation showing virtual slices through the reconstructed three-dimensional phase dataset. Within the volume, both cells' nuclei and vesicular fat metamorphosis, a pathological state of the liver, are clearly identifiable. The same volume is then showed after style transfer to H&E histology, demonstrating the microscope capability to produce non-destructive three-dimensional virtual histology of unstained tissue.

Movie S2. An animation displaying axial slices of the liver sample's region of interest (278×242 μm^2) imaged with the Nano3DX CT scanner with a pixel size of 0.63μm, corresponding to the areas presented in Figure S5. A higher density of fat vesicles is visible, however, corresponding displaced nuclei are not observed.

References

1. L Grady, Random Walks for Image Segmentation. *IEEE Transactions on Pattern Analysis Mach. Intell.* **28**, 1768–1783 (2006) Conference Name: IEEE Transactions on Pattern Analysis and Machine Intelligence.
2. M Eckermann, et al., Three-dimensional virtual histology of the human hippocampus based on phase-contrast computed tomography. *Proc. Natl. Acad. Sci.* **118**, e2113835118 (2021) Publisher: Proceedings of the National Academy of Sciences.
3. CXRO X-Ray Interactions With Matter (year?).
4. PC Diemoz, FA Vittoria, A Olivo, Spatial resolution of edge illumination X-ray phase-contrast imaging. *Opt. Express, OE* **22**, 15514–15529 (2014).
5. M Esposito, et al., A laboratory-based, low-energy, multi-modal x-ray microscope with user-defined resolution. *Appl. Phys. Lett.* **120**, 234101 (2022).
6. T Tsukamoto, et al., Micro-Computed Tomography Based Whole Block Imaging of Asthma-Associated Airway Remodeling With Mycobacterium avium-Induced Cavity Formation: 3-Dimensional Nondestructive Analysis. *Pathol. Int.* **n/a** (year?) _eprint: https://onlinelibrary.wiley.com/doi/pdf/10.1111/pin.70074.
7. JY Zhu, T Park, P Isola, AA Efros, Unpaired Image-To-Image Translation Using Cycle-Consistent Adversarial Networks. pp. 2223–2232 (2017).
8. M Gadermayr, et al., Generative Adversarial Networks for Facilitating Stain-Independent Supervised and Unsupervised Segmentation: A Study on Kidney Histology. *IEEE Transactions on Med. Imaging* **38**, 2293–2302 (2019).
9. Td Bel, M Hermsen, J Kers, Jvd Laak, G Litjens, Stain-Transforming Cycle-Consistent Generative Adversarial Networks for Improved Segmentation of Renal Histopathology in *Proceedings of The 2nd International Conference on Medical Imaging with Deep Learning.* (PMLR), pp. 151–163 (2019) ISSN: 2640-3498.

10. AC Ruifrok, DA Johnston, Quantification of histochemical staining by color deconvolution. *Anal. Quant. Cytol. Histol.* **23**, 291–299 (2001).
11. CK Hagen, FA Vittoria, ORi Morgó, M Endrizzi, A Olivo, Cycloidal Computed Tomography. *Phys. Rev. Appl.* **14**, 014069 (2020).
12. G Lioliou, et al., Nyquist-compliant cycloidal computed tomography. *Phys. Rev. Appl.* **22**, 034011 (2024) Publisher: American Physical Society.
